# Expert panel process to optimise the design of a randomised controlled trial in chronic rhinosinusitis (the MACRO programme)

**DOI:** 10.1186/s13063-019-3318-3

**Published:** 2019-04-23

**Authors:** Helen Blackshaw, Jane Vennik, Carl Philpott, Mike Thomas, Caroline Eyles, James Carpenter, Caroline S. Clarke, Steve Morris, Anne Schilder, Valerie Lund, Paul Little, Stephen Durham, Spiros Denaxas, Elizabeth Williamson, David Beard, Jonathan Cook, Steffi Le Conte, Kim Airey, Jim Boardman, Claire Hopkins

**Affiliations:** 1 evidENT, Ear Institute, University College London, London, UK; 2 Primary Care and Populations Sciences, Faculty of Medicine, University of Southampton, Southampton, UK; 3 Norwich Medical School, University of East Anglia, Norwich, UK; 4 James Paget University Hospital NHS Foundation Trust, Norwich, UK; 5 Farr Institute, University College London, London, UK; 6 London School of Hygiene and Tropical Medicine, London, UK; 7 Research Department of Primary Care and Population Health, University College London, London, UK; 8 Department of Applied Health Research, University College London, London, UK; 9 Faculty of Medicine, Imperial College London, London, UK; 10 Surgical Interventional Trials Unit, University of Oxford, Oxford, UK; 11 Guy’s and St. Thomas’ NHS Foundation Trust, London, UK; 12 Fifth Sense, Sanderum House, 38 Oakley Road, Chinnor, Oxfordshire OX39 4TW UK

**Keywords:** Expert panel, Mixed methods, Consensus, Decision-making, Chronic rhinosinusitis

## Abstract

**Background:**

MACRO (Defining best Management for Adults with Chronic RhinOsinusitis) is an NIHR-funded programme of work designed to establish best practice for adults with chronic rhinosinusitis (CRS). The 7-year programme comprises three consecutive workstreams, designed to explore NHS care pathways through analysis of primary and secondary data sources, and to undertake a randomised controlled trial to evaluate a longer-term course of macrolide antibiotics and endoscopic sinus surgery for patients with CRS. A number of outstanding elements still required clarification at the funding stage. This paper reports an expert panel review process designed to agree and finalise the MACRO trial design, ensuring relevance to patients and clinicians whilst maximising trial recruitment and retention.

**Methods:**

An expert panel, consisting of the MACRO Programme Management Group, independent advisors, and patient contributors, was convened to review current evidence and the mixed-method data collected as part of the programme, and reach agreement on MACRO trial design. Specifically, agreement was sought for selection of macrolide antibiotic, use of orally administered steroids, inclusion of CRS phenotypes (with/without nasal polyps), and overall trial design.

**Results:**

A 12-week course of clarithromycin was agreed as the main trial comparator due to its increasing use as a first- and second-line treatment for patients with CRS, and the perceived need to establish its role in CRS management. Orally administered steroids will be used as a rescue medication during the trial, rather than routinely either pre or post trial randomisation, to limit any potential effects on surgical outcomes and better reflect current UK prescribing habits. Both CRS phenotypes will be included in a single trial to ensure that the MACRO trial is both pragmatic and generalisable to primary care. A modified, three-arm trial design was agreed after intense discussions and further exploratory work. Inclusion criteria were amended to ensure that the patients recruited would be considered eligible for the treatment offered in the trial due to having already received appropriate medical therapy as deemed suitable by their ENT surgeon. A proposed 6-week run-in period prior to randomisation was removed due to the new criteria prior to randomisation.

**Conclusion:**

The expert panel review process resulted in agreement on key elements and an optimal design for the MACRO trial, considered most likely to be successful in terms of both recruitment potential and ability to establish best management of patients with CRS.

## Introduction

MACRO (Defining best Management for Adults with Chronic RhinOsinusitis) is an National Institute for Health Research (NIHR)-funded programme of work designed to establish best practice for adults with chronic rhinosinusitis (CRS) [1]. CRS is a chronic inflammatory condition of the nose and paranasal sinuses, with symptoms such as nasal congestion, nasal discharge, facial pain/pressure, and anosmia which significantly impact on a patient’s quality of life [2, 3]. Prevalence rates of CRS reach 10% in the UK adult population [4]. Whilst many patients are managed in the community, there is significant onward referral to specialist ear, nose, and throat (ENT) services. This results in up to 120,000 outpatient consultations and in excess of 40,000 surgical procedures for CRS in England and Wales annually. There are, as yet, no National Institute for Health and Care Excellence (NICE) guidelines for managing patients with CRS. The European Position Paper (EPOS) [5] provides recommendations, but it is unclear how widely they are known about or used in UK clinical practice. The MACRO programme is a 7-year programme of work which sets out to help to formulate new recommendations for CRS patient management across primary and secondary care. The MACRO programme has been designed by a multidisciplinary team including ENT specialists, general practitioners (GPs), health economists, statisticians, qualitative researchers, and experienced clinical triallists from across the UK. It is divided into three consecutive workstreams with the following aims and objectives:

- *Workstream 1*: To explore current National Health Service (NHS) care pathways for adults with CRS through: (1) analysis of routine data from health records, (2) cost analysis of care pathways, and (3) qualitative analysis of stakeholder views of current management
- *Workstream 2*: To evaluate the effectiveness of endoscopic sinus surgery (ESS) and long-term antibiotic treatment for patients with CRS, including those with (CRSwNP) and without (CRSsNP) nasal polyps
- *Workstream 3*: To combine outcomes from the workstreams 1 and 2 to define the best care pathways for adults with CRS

**Fig. 1:**
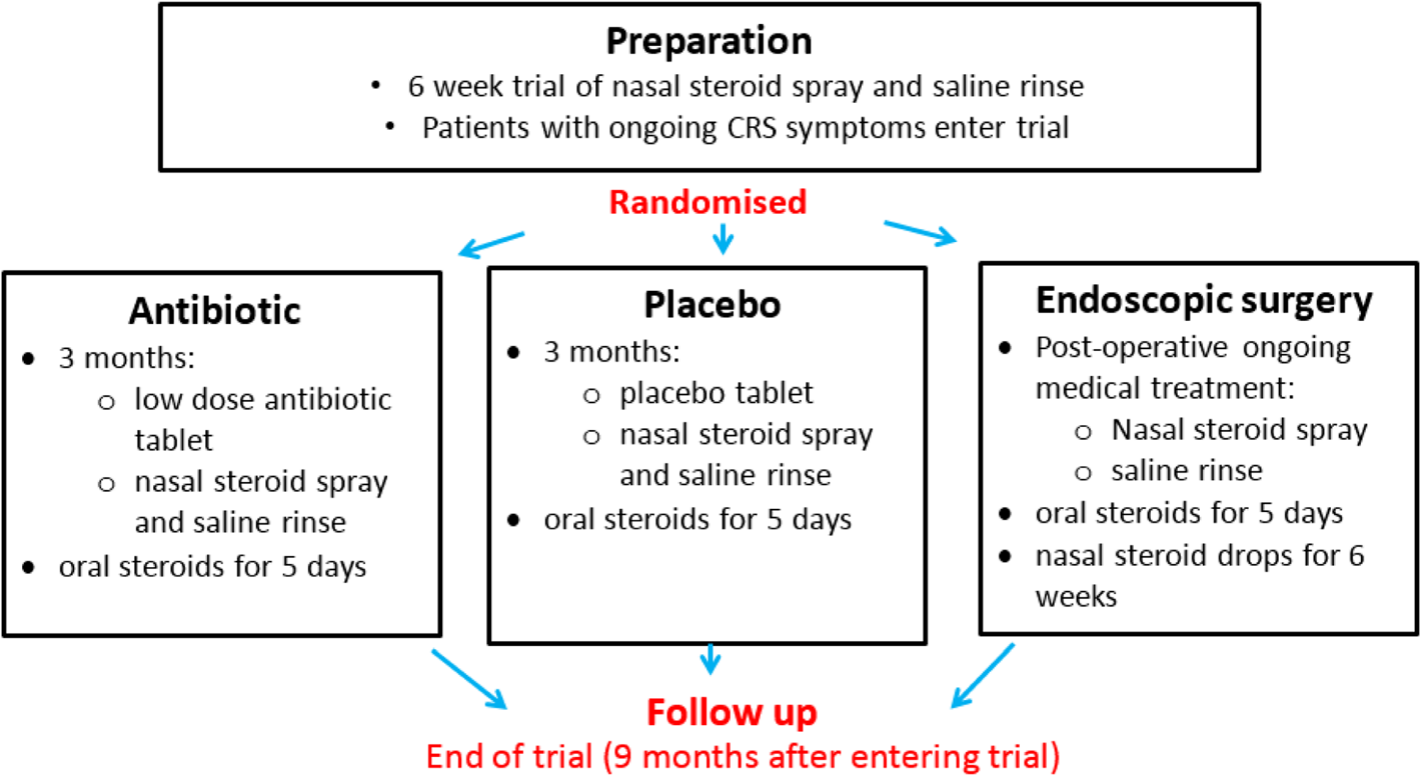
Single-stage, three-arm trial design

**Fig. 2:**
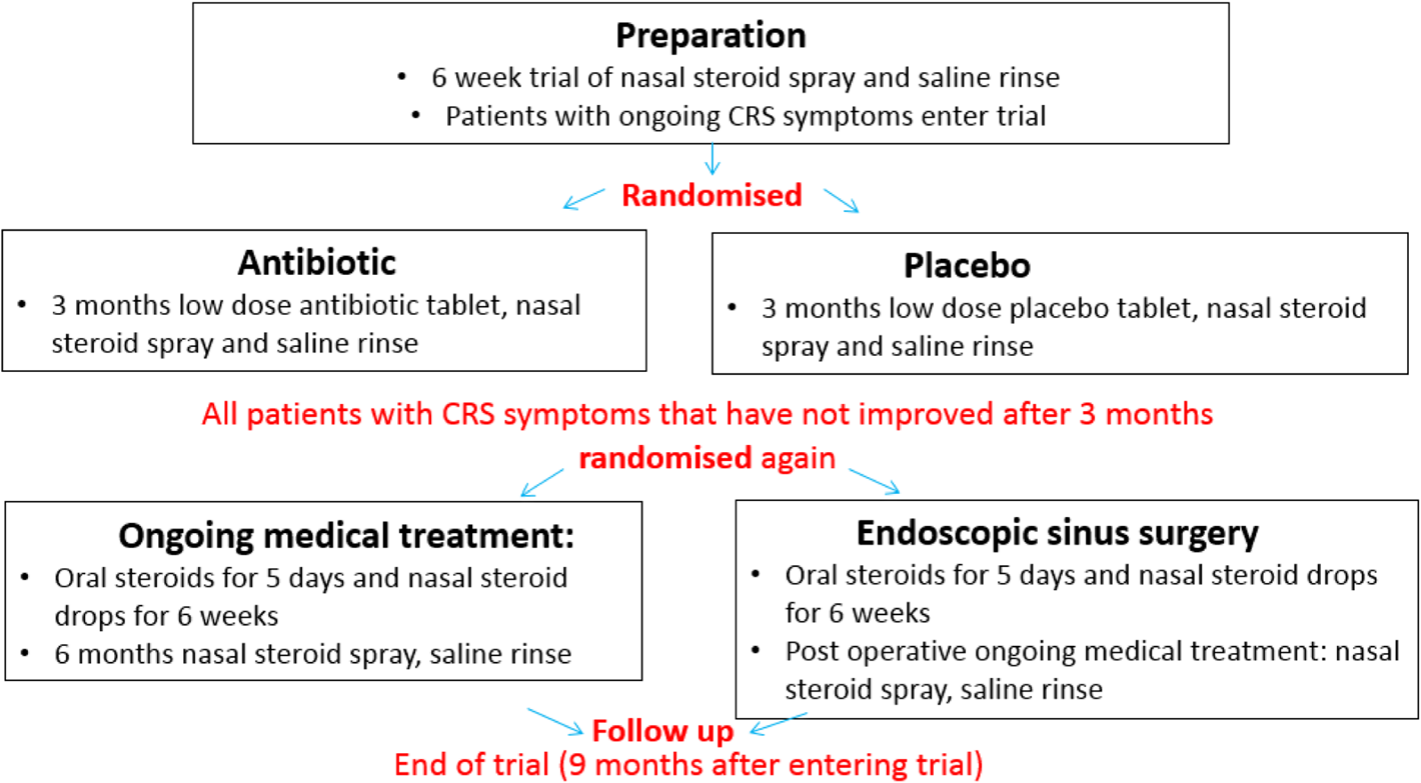
Two-stage, two-comparison trial design

National Institute for Health Research (NIHR) programme grants are aimed at providing research for the benefit of patients, public, and the NHS. There is an emphasis on multidisciplinary approaches and collaboration to ensure that the research is both relevant and applicable [1]. Within a programme of work there are commonly decision or ‘*stop-go*’ points which need answering before work continues. In the MACRO programme, the NIHR funding panel identified three key issues that required resolution prior to commencing workstream 2. These included: (1) the commitment to the use of clarithromycin as the trial comparator; (2) the use of orally administered corticosteroids (OCS) pre or post trial randomisation, and (3) possible differences in the natural history, and hence management, of CRS with and without nasal polyps. Additionally, two trial designs were proposed by the research team to address the research question regarding the effectiveness of long-term antibiotics and ESS for patients with CRS. Both were considered to be equally valid, but there were uncertainties about how each design might be implemented in practice, the acceptability to patients, and potential implications for patient recruitment and retention. Design 1 consisted of a single-stage, three-arm trial (*ESS vs macrolide vs placebo*) (Fig. 1), whilst Design 2 consisted of a two-stage, two-comparison trial (stage 1: *macrolide vs placebo,* followed by stage 2: *ESS vs ongoing medical management for symptomatic patients*) (Fig. 2).

It was agreed with the NIHR programme panel that the results of workstream 1 would be used to determine the key elements of the final trial design, using a multidisciplinary and collaborative approach. This paper reports the process of convening an expert panel of researchers, methodologists, triallists, and public contributors to review and integrate current evidence with the mixed-method data collected in workstream 1. The aim of this process was to address and resolve the following outstanding trial elements:

i. To justify and commit to the use of clarithromycin in the MACRO trial
ii. To agree on the role of OCS both pre and post randomisation to the MACRO trial
iii. To review treatment and management pathways for CRSwNP and CRSsNP patients, and agree how best to evaluate both phenotypes in the trial
iv. To agree on the final MACRO trial design.

## Methods

### Study design

This is an expert panel review of current evidence and mixed-method data to reach agreement on key elements of the MACRO trial design, as part of a decision-making process in an NIHR programme of work in CRS.

### Expert panel

An expert panel was convened by the MACRO programme manager (HB) on 12 July 2017 at the Royal College of Surgeons, London. The panel included: (1) members of the MACRO Programme Management Group (PMG) (MACRO chief investigators, co-investigators across the specialist areas, the programme manager and a patient contributor); (2) researchers and operational teams involved in workstreams 1 and 2; and (3) independent contributors including a professor of medicine, a qualitative researcher and a patient and public contributor, recruited through personal contact (clinician and researcher) and through the Fifth Sense charity (patient). The role of the independent contributors was to provide an impartial view to the review of the research data and help the team to reach agreement. Each independent contributor was asked to sign a confidentiality agreement prior to the expert panel meeting in light of the pre-publication research data. Prior to the meeting, all panel members received an information pack detailing the background and aims of the MACRO programme, the purpose of the expert panel meeting, and the role of invited independent members. The meeting was chaired by Professor Claire Hopkins, joint chief investigator of the MACRO programme, and lasted for a total of 4 h.

### Data collection

#### Quantitative data

Quantitative observational cohort data were obtained from the Clinical Practice Research Datalink primary care database, the Hospital Episode Statistics (HES) hospital care database in England, and the Office for National Statistics mortality dataset. Linked electronic health records from these sources were examined through the CALIBER platform. Access was granted following an Independent Scientific Advisory Committee (ISAC) application (protocol number 16_200R). Primary care data were extracted from the Clinical Practice Research Datalink (CPRD) Gold (January 2017 version). Hospital Episode Statistics data were obtained for CPRD-linked patients, with coverage up to 29 February 2016. Office of National Statistics death data were obtained for CPRD-linked patients, with coverage up to 8 March 2016. Patients with a diagnostic term of polyps in the HES data for surgery provided a level of certainty of phenotype diagnosis. However, recording of polyp status in secondary care data is poor, and there are diagnostic uncertainties in primary care due to limited specialist equipment. The data are, therefore, presented as ‘*polyps present*’ (requiring surgery) and ‘*polyps unknown*’ (likely to include those with less severe polyps not requiring surgery, together with those without polyps).

#### Qualitative data

Qualitative data were collected via semi-structured telephone interviews with a purposeful sample of 12 GPs, nine ENT specialists and 25 patients with CRS recruited through primary and secondary care between January and April 2017. Ethical approval was given by the Health and Social Care Research Ethics Committee A on 22 September 2016 (16/NI/0197) and all participants gave their informed consent prior to the interview. Healthcare professionals were asked about their views of diagnosis and management of CRS, knowledge and implementation of guidelines, and perceptions of the evidence base. Patients were asked about their experiences of living with CRS, and views of the different treatment options available in primary and secondary care. All participants were presented with the two trial designs (Figs. 1 and 2) prior to the interview and asked their views and opinions about the barriers and facilitators to each design, with respect to treatment arms, timings of treatments, recruitment of patients and retention in the trial. Further details about recruitment and data collection are published elsewhere [6].

### Analysis

Qualitative and quantitative data were individually analysed in three separate analyses: health informatics (EW, SD, JC), health economics (CC, SM), and qualitative (JV, CE, MT). The methods and results of these individual analyses are presented independently elsewhere [6]. The expert panel convened in July 2017. Prior to the meeting, panel members received information about the MACRO programme, including details about the three workstreams and research methods used in workstream 1. During the meeting, the joint chief investigators (CH and CP) presented background to the MACRO programme, the recent literature and outlined the specific aims and objectives of the panel meeting. The four key aims were addressed in turn. In each case, a review of the current evidence was presented (CH). The results of the individual analyses were then presented: health informatics, qualitative work, and health economics. The data were then discussed and debated, with areas of agreement, partial agreement, and disagreement identified. Each member had the opportunity to ask questions and contribute to the discussions. Resolution for each key point was summarised and proposed by the chair (CH) and voted on with a ‘show of hands’ by the panel members. All members’ responses carried equal weight, and consensus was determined by majority view. Where possible, discussions continued until agreement was reached. In the case where discussions did not lead to a majority view, a post-panel meeting plan of action was agreed.

## Results

### Selecting the appropriate macrolide antibiotic

#### Current evidence

Macrolide antibiotics have been shown to have anti-inflammatory and immunomodulatory properties, reducing the pro-inflammatory cytokines critical in CRS [7, 8]. International guidelines recommend the use of low-dose, long-term macrolides in selected patients with CRS but the evidence is weak, and also based on roxithromycin which is not currently available in the UK [9, 10]. A survey of 158 UK ENT surgeons (2013) [11] found that 92% prescribed antibiotics prior to ESS surgery, with 16% prescribing 12 weeks’ macrolide treatment. The UK sinonasal audit [12] in 2000 found that 7% of CRSwNP and 18.9% of CRSsNP patients received low-dose, long-term antibiotics prior to ESS.

Treatment duration has been evaluated in some low-quality studies, but the evidence suggests that longer courses of macrolide therapy are more effective than shorter courses in patients with CRS [13, 14].

More recently, concerns have been raised about potential cardiovascular risks from the use of full-dose, short-term macrolides [15]. Macrolides may cause prolongation of the QT interval which can lead to arrhythmia and myocardial infarction. However, the safety of lower doses for longer periods in such cases for CRS has yet to be investigated.

#### Health informatics

The health informatics analysis evaluated current trends in antibiotic prescribing, including treatment duration and safety. The analyses found that penicillins remain the first-choice antibiotic for patients with a high likelihood of CRS; however, a recent trend towards the increased use of macrolides and tetracyclines (mainly doxycycline) was found for patients with a definitive diagnosis of CRS.

The most common treatment duration for both macrolide and penicillin prescriptions for CRS was found to be 1 week. However, macrolides were likely to be prescribed for a longer durations (over 8 weeks) than penicillins, particularly for those with a definitive CRS diagnosis.

Analysis of macrolide safety data found that there was no statistically significant short- or long-term risk associated with macrolide prescription compared to penicillin for patients with CRS. A potential (non-significant) short-term risk of myocardial infarction during the first 30 days following prescription was found with macrolide antibiotics. However, no significant risks were particularly associated with clarithromycin, and there was no evidence of increased risk of cardiovascular events.

#### Qualitative findings

GPs described prescribing short courses of antibiotics in primary care, with limited experience of long-term antibiotic use for patients with CRS. ENT surgeons commonly described the use of clarithromycin in patients prior to sinus surgery; however, treatment durations were often less than 8 weeks, and generally for CRSwNP patients. Patients described the role of antibiotics as appropriate to treat infections and were uncertain about their use in CRS. Some expressed concerns about potential gastrointestinal side effects and antibiotic resistance, especially with longer courses of treatment.

#### Expert panel review

The expert panel agreed that clarithromycin should be selected as the macrolide of choice for the MACRO trial due to its increasing use as a first- and second-line treatment for patients with CRS, and the perceived need to establish its role in CRS management. A 12-week course of treatment was selected as the appropriate treatment duration based on the evidence for effectiveness with longer treatment durations and no associated increased risk with longer treatment courses. To mitigate against any potential problems associated with increased cardiovascular risk with macrolide treatment, the panel agreed that patients with a prior history of ischaemic heart disease and those aged over 65 years with diabetes will be excluded from the MACRO trial. A baseline electrocardiogram (ECG) will be added to the trial screening process to identify and exclude those with prolongation of the QT interval.

### Role of orally administered corticosteroids (OCS)

#### Current evidence

Short courses of OCS are commonly used in treating patients with CRS, either alone or in combination with other treatments. Currently, international guidelines (EPOS [5]) recommend OCS for severe CRSwNP patients. A Cochrane review [16] suggests that a short course of OCS improves quality of life and symptom control compared to placebo or no treatment for patients with nasal polyps, although little or no difference was noted after 3 months. Another review found that as an adjunct treatment, OCS may result in an improvement in symptom severity and reduction in nasal polyps, but the quality of evidence was low, and no data were available to assess the longer-term benefits [17]. To date, no randomised controlled trials (RCTs) have assessed the role of OCS for CRSsNP. Prescribing of OCS for CRS has been evaluated in a number of studies. A UK survey in 2013 found that 34% of ENT surgeons sometimes or always prescribed OCS as part of maximal medical therapy for CRS [11]. A Canadian survey in 2016 found that 79% prescribed OCS for CRSwNP, whilst 23% prescribed them for CRSsNP at least sometimes [18]. The UK Sinonasal Audit in 2000 [12] found that 14% of CRS patients received OS prior to ESS (18% of CRSwNP compared to 6% of CRSsNP), suggesting that OCS use is not routine for all patients prior to ESS.

#### Health informatics

The health informatics data provided limited information about the prescribing of OCS for CRS. The CPRD only captures primary care prescriptions for CRS, whilst secondary care prescribing of OCS is only captured if prescription advice is sent back to the patient’s GP. Data presented to the panel meeting included combined data for intranasally administered corticosteroids (INCS) and OCS. Patients with polyps present and polyps unknown were both highly likely to receive a steroid prescription (INCS or OCS) both before and after sinus surgery, but no conclusions could be drawn for the individual usage of OCS.

#### Qualitative findings

GPs reported infrequent use of OCS in primary care, with some GPs expressing a lack of confidence for use in CRSwNP. ENT specialists described the use of OCS for CRSwNP prior to surgery, and for occasional rescue courses for severe cases. However, concerns were raised about the potential of a ‘*pre-operative blast of steroids*’ to affect surgical outcomes which could consequently affect trial results. Likewise, post-operative OCS were described as ‘*muddying the waters*’, resulting in an unclear picture of the effectiveness of surgery alone. A few patients had experience of OCS use and described rapid symptom relief especially after surgery. Whilst there were some reported concerns about side effects, patients were generally agreeable to their use if recommended by their specialist.

#### Expert panel review

The expert panel agreed that there was insufficient evidence to support the routine use of OCS, either in the 3-month run-up to the MACRO trial or post randomisation. Nevertheless, it was agreed that a short course of OCS could be used as a rescue medication during the trial, whereby usage would be documented and compared between treatment arms.

### Care pathways: polyp and non-polyp patients

#### Current evidence

Care pathways for CRSsNP and CRSwNP are uncertain, but there are like to be key similarities in patient management, especially at a the primary care stage. GPs do not have access to endoscopy or computed tomography (CT) scanning, and, therefore, distinguishing between patients based on polyp status is difficult unless polyps are big enough to be visible at the nostrils. It is likely, therefore, that primary care management and referral would be similar for both phenotypes. The Chronic Rhinosinusitis Epidemiology Study found that rates of antibiotic use, OCS use, and nasal irrigation were not significantly different between the two main subgroups of CRSwNP and CRSsNP patients [19], suggesting similar treatment pathways. Ultimately, it is possible that a CRSsNP patient may subsequently develop nasal polyps, and thus converge any treatment and management pathways for CRSsNP and CRSwNP.

#### Health informatics

From CPRD data, both the ‘*polyps present*’ and the ‘*polyps unknown*’ groups showed similar patterns of consultation in primary and secondary care. Prescribing rates were broadly comparable; however, slightly higher levels of OCS prescribing were observed in the ‘*polyps present*’ group, whilst antibiotic prescribing was slightly increased when the polyp status was unknown. From the health economic analysis, where the highest CRS cost relates to ESS surgery, mean costs per patient were similar between both groups.

#### Qualitative research

ENT surgeons and GPs described some differences in the CRS patient journey for CRSwNP and CRSsNP. Patients with nasal polyps were described as difficult to diagnose in primary care due to the lack of diagnostic facilities, but patients with visible polyps were more likely to be prescribed medical treatment (steroid drops/OCS) in primary care and receive an early referral for specialist opinion. In secondary care, ENT specialists reported that patients with nasal polyps were more likely to be prescribed OCS and listed earlier for surgery.

For patients without nasal polyps, GPs described themselves as confident in making the CRS diagnosis, and management commonly included a range of INCS sprays. Onward referral was often not prioritised for non-polyp patients and patient pressure and lack of treatment response were the main drivers to secondary care referral. In secondary care, patients without nasal polyps were more likely to receive long-term antibiotics, and patient preference contributed to the decision for surgery.

#### Expert panel review

The expert panel considered the options of either conducting separate trials or including both phenotypes in a single trial. In light of the diagnostic difficulties in primary care and the possibility that CRS patients without polyps may subsequently develop them, the expert panel agreed that two separate studies would be less relevant to the real-world setting. Including CRSwNP and CRSsNP patients in a single trial was considered to be more pragmatic, and likely to provide answers that could be generalised back to primary care, where polyp status can be uncertain. It was agreed, however, that trial analysis should include stratification for polyp status to determine whether outcomes vary by CRS phenotype.

### MACRO trial design

#### Qualitative research

There was a mixed response to the two proposed trial designs both from clinicians and patients. Some clinicians expressed a preference for the single-stage, three-arm design (Fig. 1) describing it as ‘*simple to understand and describe to patients*’, potentially easier to recruit to, and evaluated surgery earlier in the patient pathway. Likewise, patients liked the simplicity of the design, but some expressed concerns that surgery was too early in their treatment journey, without all medical treatment options being explored first. However, clinicians generally considered the two-stage, two-comparison design (Fig. 2) to be a ‘*more conservative approach*’ and more closely aligned to current practice, but was potentially more complex for patients to understand. Concerns were also raised about retention of patients for the second randomisation. Patients generally liked the two-stage, two-comparison design, describing it as allowing them to try medical treatments first, with the potential for surgery to be delayed or avoided.

#### Expert panel review

The panel agreed that the qualitative work highlighted barriers and facilitators to both trial designs and debated the key issues at length. The main issues arising about the single-stage, three-arm design (Fig. 1) concerned the use of medical treatment prior to trial entry, and the potential timing of surgery. Some patients were concerned about the possibility of being randomised to surgery before all medical treatment options had been explored. This raised the question about what was considered to be ‘appropriate medical treatment (AMT)’ before a patient was deemed a suitable candidate for surgery. Guidelines suggest that AMT should include a trial of INCS for at least 8 weeks, with the optional adjunct of saline irrigation [20]. For CRSsNP patients, a short course of broad-spectrum antibiotics or low-dose macrolides is appropriate, and CRSwNP patients may benefit from a short course of OCS. Health informatics data identified that most patients receive INCS and a short course of antibiotics prior in primary care, and, therefore, were likely to have received AMT prior to consultation in secondary care where trial recruitment will take place.

Following intense debate of the key issues, the expert panel was unable to reach consensus on the trial design during the panel meeting itself. Further exploratory work was agreed to help address the key issue surrounding AMT prior to surgery, including exploring patient views of AMT prior to surgery, and trial investigator views of patient eligibility for surgery. The following work was carried out:

- Patients who participated in the original qualitative research (*n* = 25) were presented with a scenario of ‘being invited to participate in a three-arm trial if their ENT specialist felt they were eligible for surgery on the basis of already receiving AMT’. In the sample of patients who responded (*n* = 18/25), most agreed that theoretically they would consent to take part in such a trial. However, seven would not take part (four patients would prefer to choose their treatment rather than being randomised, and three expressed concerns about the risks of early surgery)
- Principal investigators (PIs) for the MACRO trial (*n* = 10) were approached to explore their attitudes to AMT, specifically the treatments that they considered essential prior to patients being eligible for surgery (and, hence, the trial). In general, INCS were described as essential for both CRSwNP and CRSsNP patients. OCS were considered essential only for polyp patients. Short-term antibiotics were often used for both phenotypes but were not generally considered to be essential. Views on the importance of prior treatment with long-term antibiotics was varied for both phenotypes. Overall, failed medical treatment was described as the main criteria for surgery

These results were presented back to the expert panel who had the opportunity to consider and review the additional information and reflect on the panel discussions and opinions of the other panel members. Each panel member was then asked to vote for their preferred trial design, by emailing their decision to the programme manager, describing the reasons for their choice. The results were collated and a trial design agreed.

**Fig. 3:**
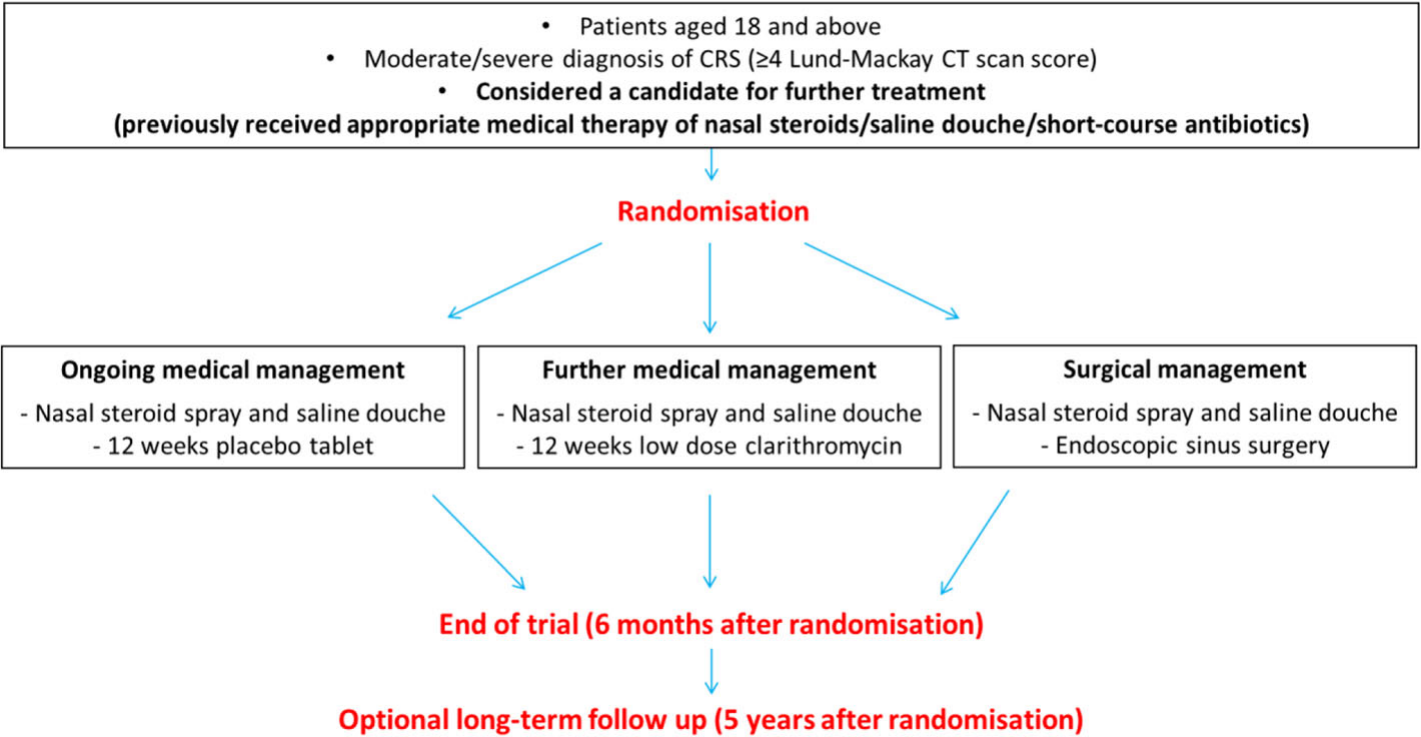
Modified, three-arm trial design

The final agreed trial design was a modified, three-arm design (Fig. 3) where patients would be eligible if the local PI considered them to be suitable candidates for further treatment (including surgery). This modified design addressed the key issues of patient concern about timing of surgery (patients will have received AMT prior to being considered eligible for the trial) and AMT (practice is varied but most patients will have received antibiotics and INCS in primary care, although macrolide treatment is not widely used).

## Discussion

### Summary

An expert panel review of the best available evidence and mixed-method data resulted in agreement on key elements and optimal design for the MACRO trial, considered most likely to be successful in terms of both recruitment potential and ability to establish best management of patients with CRS. A 12-week course of clarithromycin was agreed as the main non-surgical intervention, due to its increasing use as a first- and second-line treatment for patients with CRS, and the perceived need to establish its role in CRS management. The control arm will receive placebo. Orally administered corticosteroids will be used as a rescue medication during the trial, rather than routinely either pre or post trial randomisation, to limit any potential effects on surgical outcomes and better reflect current UK prescribing habits. Both CRS phenotypes will be included in the same trial to ensure the MACRO trial is both pragmatic and generalisable. A modified, three-arm design was agreed (Fig. 3). Inclusion criteria were amended to ensure that the patients recruited into the trial would be eligible for ESS (with a Sino-nasal outcome test (SNOT-22) score of ≥ 20, evidence of CRS on a recent CT scan), and have received appropriate medical therapy (AMT) as deemed suitable by their ENT surgeon. The proposed 6-week run-in period prior to randomisation was removed due to new criteria for AMT prior to randomisation.

### Strengths and limitations

The expert panel process resulted in optimisation of the final MACRO trial design using a multidisciplinary and collaborative approach, ensuring that the agreed trial design was both relevant and appropriate, and based on best available evidence.

All members of the panel engaged fully with the expert panel process and were committed to reaching consensus on all elements of the trial design. Independent members contributed equally to the discussions and ensured that the evidence and data were reviewed in a balanced and impartial way.

There were a number of challenges associated with conducting this expert panel review. A large amount of varied and complex data were produced as part of this work which needed summarising in such a way as to be relevant and coherent to a range of expert panel members. Panel members needed to be sufficiently prepared to review and debate the evidence and integrate the data, with the aim of reaching agreement.

The panel consisted of members with different expertise, backgrounds and interests, and often views were conflicting. It was not possible to reach final agreement for the overall trial design during the panel meeting itself. Panel members needed additional time for consideration of the data presented and reflect on discussions and opinions from the individual experts and patient contributors before consensus was reached.

### Expert panel process

- The use of an expert panel process is a valid and valuable method for addressing decision or ‘*stop-go*’ points in a research programmes
- When decisions are potentially complex, a multidisciplinary and collaborative approach ensures that the issues have been discussed and debated in detail, and outcomes are relevant and considered
- Panel members’ views are often divergent, but it is important to listen to and consider the individuals’ views and opinions as all are equally important and valid
- Research teams need to remain open to all possible outcomes of the panel review, without having preconceptions or expectations
- When important decisions impact widely on a research programme, sufficient time should be allocated for individual reflection, as well as group discussions. A two-stage process should be considered if time permits

## Conclusion

This expert panel review process has successfully resulted in the production of a modified trial design deemed most likely to be successful in terms of both recruitment potential and ability to establish best management of patients with CRS. The MACRO trial protocol has received ethical and governance approvals, and the study opened to recruitment in November 2018.

## Abbreviations

AMT: Appropriate medical therapy
CPRD: Clinical Practice Research Datalink
CRS: Chronic rhinosinusitis
CRSsNP: Chronic rhinosinusitis without nasal polyps
CRSwNP: Chronic rhinosinusitis with nasal polyps
CT: Computerised tomography
ECG: Electrocardiogram
ENT: Ear, nose, and throat
EPOS: European Position Paper
ESS: Endoscopic sinus surgery
GP: General practitioner
HES: Hospital Episode Statistics
INCS: Intranasally administered corticosteroids
MACRO: Defining best Management for Adults with Chronic Rhinosinusitis
NHS: National Health Service
NICE: The National Institute for Health and Care Excellence
NIHR: National Institute for Health Research
OCS: Orally administered corticosteroids
PGfAR: Programme Grants for Applied Research
PMG: Programme Management Group
RCT: Randomised controlled trial
SNOT-22: Sino-nasal outcome test
UK: United Kingdom
